# Expanding the UTAUT2 framework to determine the drivers of mobile shopping behaviour among older adults

**DOI:** 10.1371/journal.pone.0295581

**Published:** 2023-12-14

**Authors:** Tianyang Huang

**Affiliations:** School of Mechanical Engineering, Guangdong Ocean University, Zhanjiang, China; Al-Ahliyya Amman University, JORDAN

## Abstract

In the current severe aging of the population, the problem of "digital divide" of the elderly has become increasingly prominent, and the elderly market represents a vast demographic that is increasingly becoming an important customer segment for mobile shopping in the future. However, there is currently insufficient attention given to the research on mobile shopping behavior among older adults. This study tries to answer what are the driving factors of mobile phone shopping behavior among the elderly? The purpose of this study is to analyze the factors that drive the elderly’s mobile phone shopping behavior, and to establish a mobile phone shopping acceptance model for the elderly to predict the factors of the elderly’s behavioral intention of using smart phones. Based on the second edition of Unified Theory of Acceptance and Use of Technology theory (UTAUT 2), this study proposed a mobile phone shopping acceptance model for the elderly. The study collected valid data from 389 Chinese elderly people through questionnaires and analyzed them using structural equation models. The results showed that utilitarian, anxiety, trust, performance expectancy, effort expectancy, social influence, facilitating conditions and habit directly impact the older adults’ intention to engage in mobile shopping. Additionally, facilitating conditions, habit and the older adults’ intention to engage in mobile shopping act as driving factors for actual use behavior. This study further expands the UTAUT theoretical model, provides a theoretical basis for the research of mobile shopping behavior of the elderly, and enricues the application groups and fields of the UTAUT theoretical model. The results of this study provide inspiration for the development, design and marketing of age-appropriate mobile shopping products, and contribute to the realization and further adoption of age-appropriate mobile shopping, and also contribute to promoting the active aging of the elderly.

## Introduction

With the development of the internet and smart technology, digital shopping and mobile applications have become increasingly abundant, thereby stimulating people’s mobile shopping activities [1]. Traditional mobile devices primarily featured call and text messaging functions [2]. However, with technological advancements, mobile phones have evolved into multimedia products for interactive communication [3]. The flexibility, mobility, and personalization of mobile phones distinguish mobile shopping from traditional e-commerce or offline stores [4]. Simultaneously, the changing user demands have brought about numerous mobile business opportunities [5], such as mobile shopping [6] and mobile banking [7]. Saprikis et al. [8] pointed out that as the integration of mobile technologies, such as mobile usage and mobile payments, continues to expand, mobile shopping consumers will persist and gradually increase. In the future, the number of mobile users conducting shopping activities through their mobile devices will significantly rise [8]. This trend underscores the necessity for user adoption research in the realm of mobile commerce [8]. However, Sanakulov and Karjaluoto [9] point out that although new smartphone devices have many services, this does not mean that users will immediately use existing mobile services, so it is necessary to explore the reasons behind this.

In reality, due to the existence of numerous potential challenges, the adoption of mobile devices exhibits considerable uncertainty [10]. Parker and Wang [11] noted that user motivations and behaviors differ when using mobile devices compared to desktop computers, due to variations in product context and constraints. Previous research has predominantly focused on users from technologically advanced developed countries within the context of e-commerce. For instance, Omar et al. [12] conducted their study in the United Kingdom, while Morosan [13] focused on American consumers, and Escobar-Rodríguez and Carvajal-Trujillo [14] targeted Spanish users. Additionally, previous studies have explored impulse buying among millennials [15], luxury purchases [16], and green purchases [17]. However, considering the fact that the current reality of aging populations, greater attention needs to be given to the vast user segment of older adults. According to a report released by World Health Organization (WHO) [18], the population over 65 years old is growing faster than the population under 65 years old. The report projects that the world’s population will reach 8 billion by November 15, 2022, and that the global population will grow to around 8.5 billion by 2030, 9.7 billion by 2050 and 10.4 billion by 2100. The share of the global population aged 65 and over will rise from 10 per cent in 2022 to 16 per cent in 2050, and by 2050 there will be more than twice as many people aged 65 and over as there are children under five. According to the data of the National Bureau of Statistics of China [19], by the end of 2022, the elderly population aged 60 and above in China has reached 280.04 million, accounting for 19.8% of the total population, and the elderly population aged 65 and above has reached 209.78 million, accounting for 14.9% of the total population. As of June 2023, the number of online shopping users in China reached 884 million, an increase of 38.8 million compared with December 2022, accounting for 82.0% of the total Internet users [20]. It can be said that as the elderly population continues to grow, their online presence and activity will increase, positioning them as crucial potential customers for future online shopping services [21]. Today, more and more industries are gradually recognizing the importance of elderly users as a potential market, but few online shopping services have taken the elderly into consideration when designing, and there is currently a scarcity of research on the online shopping behavior of older adults [21]. Most e-commerce industries fail to recognize the business opportunities brought by this growing potential customer market of older users [22], and research specifically targeting older users holds significant potential [23]. To sum up, mobile shopping behaviors of elderly users need to be studied urgently. Therefore, this study focuses on investigating older adult users within the context of mobile shopping.

Mobile shopping channels have become crucial mediums connecting users’ product purchases and merchants [24]. The popularity of smartphones and tablets has contributed to the rapid development of mobile shopping [8]. The popularity of mobile commerce in daily life has become one of the ways people search and purchase products and services online [25, 26]. Iglesias-Pradas and Pascual-Miguel [27] point out that understanding the drivers of mobile commerce user behavior is critical to the growth of e-commerce. Understanding the factors influencing mobile commerce adoption among mobile users can assist relevant mobile business enterprises in formulating or altering their business strategies [8], so as to improve users’ satisfaction with mobile services. Al-Adwan and Alrousan [28] set up a willingness model for Jordanian undergraduates to shift from traditional e-commerce to mobile commerce. The results show that the perceived technology and value differences can explain consumers’ mobile commerce intentions. Al-Adwan and Al-Debei [29] investigated the factors affecting users’ e-commerce buyback and positive word-of-mouth intentions, and the results showed that consumer behavior factors represented by customer trust and customer satisfaction would affect users’ re-purchase and positive word-of-mouth intentions. Currently, certain theories are applied as theoretical foundations in mobile shopping research, exploring factors such as perceived usefulness, satisfaction, convenience, and perceived ease of use on users’ technological behaviors. For example, Davis [30] proposed the Technology Acceptance Model (TAM), the Theory of Planned Behavior (TPB) [31], and the Unified Theory of Acceptance and Use of Technology (UTAUT) [32]. In addition, Al-Adwan et al. [33] used signaling theory and relational signaling theory to explain the online purchase intention of Jordanian users. The Unified Theory of Acceptance and Use of Technology (UTAUT) put forth by Venkatesh, Morris, Davis, and Davis [32] is a well-known theory in user behavior research. This theory integrates relevant elements from eight previous user acceptance models, providing a rich theoretical foundation. Consequently, UTAUT has been widely applied in studies of technology adoption by users, such as mobile applications [2] and mobile banking [34]. At the same time, UTAUT’s high explanatory power has led to its widespread attention and use, making it one of the most concise and predictive theoretical models for predicting consumer technology use [35], and it has proven effective in many different cultural contexts [36].

Although Venkatesh et al. [32] believe that the UTAUT model is complete and universally applicable, Heerink [37] points out that it still needs to be further adjusted to be applicable to studies in different environments. Subsequently, Venkatesh et al. [35] introduced three additional constructs and developed the UTAUT2 model. In the context of e-commerce, UTAUT2 has demonstrated strong explanatory power, for instance, in online shopping [38] and mobile commerce [39]. However, Park [40] argued that although the UTAUT model is concise and popular, its limitation lies in consolidating numerous theories and models into a single research framework. Furthermore, despite the improved explanatory power of the UTAUT2 model, it has not been widely adopted [41]. Considering the added constructs in the UTAUT2 model, namely hedonic motivation, price value, and habit, which are highly relevant to mobile shopping [35], this study employs UTAUT2 as the theoretical foundation to explore the motivating factors of mobile shopping behavior among older adults. Of course, we believe that UTAUT2 is more suitable for this study context because it was specifically developed from the user’s point of view [35]. UTAUT2 extends the applicability of UTAUT from the overall organizational context to the individual consumer context [42]. As Tamilmani et al. [43] pointed out, it is important to extend the UTAUT model and UTAUT2 by including other relevant variables in future studies. In summary, in view of the important role of UTAUT2 in the user adoption environment, this study uses UTAUT2 as the main theoretical basis to clarify the determinants of elderly consumers’ shopping intentions when using mobile phones. In addition, it should be noted that in the expansion of UTAUT, the original moderating factors can be deleted, because the model with the removal of the modulator is more able to be not limited to a specific context [44], and a more concise model can be constructed, focusing on the relationship between various constructs [45, 46]. Just as Hujran and Al-Debei [47] remove the moderating variables in UTAUT in the study on the use of intelligent government, the exploration of driving factors for the use of intelligent government will not be affected. Therefore, moderating variables such as demographic characteristics of users were not taken into account in this study. Some previous studies [48, 49] pointed out that utilitarian and hedonistic values are important driving factors of consumer purchasing behavior. However, the extent to which these variables predict mobile shopping behavior has not received enough attention [50]. Therefore, it is necessary to incorporate utilitarianism and other variables into relevant theories to better understand the consumer behavior of users. In addition, considering the extremely personalized characteristics of mobile devices, people’s concerns about privacy and security are self-evident. Lu and Su [51] pointed out that it is an important issue to study whether users’ negative reactions will have an impact on users’ technology adoption. Therefore, this study also uses anxiety as an extended variable of the UTAUT2 model to gain an in-depth understanding of users’ shopping behavior.

Thanks to advancements in Information and Communication Technology (ICT) and the widespread use of mobile devices, mobile shopping has gained increasing attention in recent years [8]. However, despite the growing trend of mobile shopping, achieving a comprehensive understanding of it remains a goal for researchers and practitioners, and the current research on mobile shopping behavior is still insufficient [52]. Zhong [52] pointed out that most of the previous studies focused on general mobile services, with little research on mobile shopping. As Chopdar et al. [53] pointed out, research on mobile commerce is still in its early stages, with a primary focus on user characteristics in technology adoption studies. Furthermore, existing studies mostly target young people, including college students, and often consider young people as the primary market for Information and Communication Technology (ICT) adoption [54]. These studies mainly concentrate on young individuals and gather research samples from student populations. However, although more and more industries today are gradually recognizing the importance of older users as a potential market, there is still a lack of attention to older adults’ shopping behavior [21]. In addition, as Al-Adwan, et al. [29] points out, most current studies are limited because they tend to focus on basic characteristics such as the personal, psychological, and cognitive properties of e-commerce. Considering that user motivation is an important component of user behavior [55], and with the substantial market of older adults who are becoming significant potential customers for online shopping services in the future [21], there is an urgent need to understand the factors influencing older adults’ use of mobile shopping. This study tries to answer what are the driving factors of mobile phone shopping behavior among the elderly? More specifically, this study aims to build a framework to explain the drivers of mobile shopping behavior in older adults. Therefore, this study examines the motivations behind older adults’ mobile shopping behavior. Based on the UTAUT2 theory, the study attempts to propose an expanded UTAUT2 model to understand the intention and usage behavior of older adults in mobile shopping. The research sample consists of older adults from a developing country, China, and the structural equation modeling is applied for validation. This study provides a theoretical basis for exploring the mobile shopping behavior of older users, contributing to the realization and further adoption of age-friendly mobile shopping.

This study has some contributions. First, this study incorporating Utilitarian, Anxiety, and Trust factors in the context of mobile shopping, confirming the applicability of the extended UTAUT model in researching mobile shopping among older adults, And demonstrated how Utilitarian, Anxiety and Trust factors affect the mobile phone shopping behavior of elderly people. Second, this study proposes a model for the acceptance of mobile shopping by older adults, study extends the UTAUT theoretical model. This provides a theoretical foundation for researching older adults’ mobile shopping behavior. Third, this research was conducted in China, the world’s largest consumer market, which differentiates it from previous studies that primarily focused on samples from developed countries [13, 14]. and younger generations [15], which enriches the applicable objects of the UTAUT theoretical model. Lastly, the study can provide reference for mobile shopping practitioners and policy makers to develop relevant marketing and management strategies to increase users’ adoption of mobile shopping. It also provides inspiration for the development, design, and marketing of age-friendly mobile shopping products, thereby enhancing positive aging among older adults.

## Theory and research hypothesis

**Fig 1:**
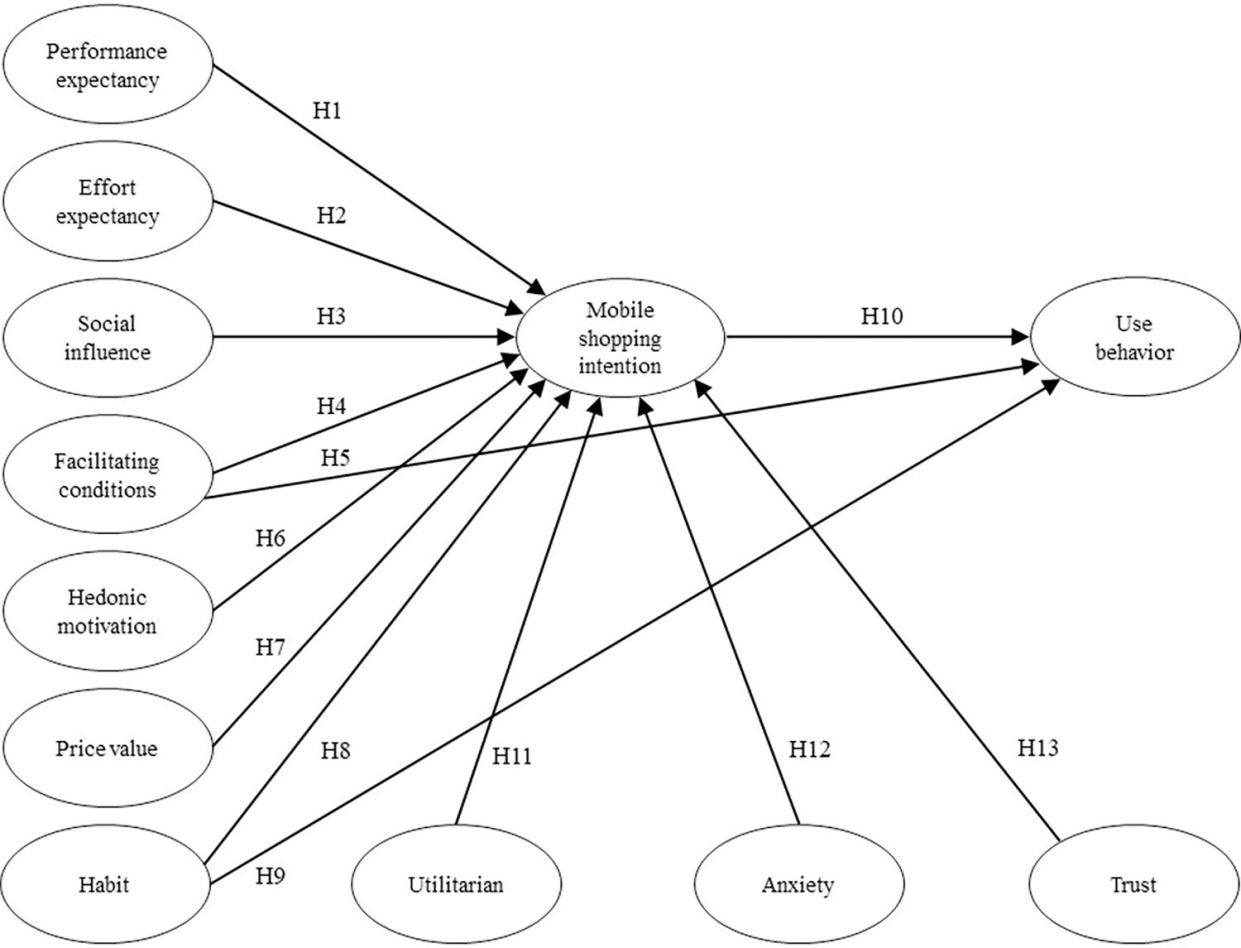
The research model.

On the basis of literature review, this section explains the variables, proposes relevant hypotheses based on previous literature, and builds a conceptual model of mobile shopping acceptance for the elderly (Fig 1). This conceptual model consists of 7 variables in UTAUT 2, including Performance Expectancy, Effort Expectancy, Social Influence, Facility Conditions, Hedonic Motivation, Price value, Habit, Behavioral Intention, and 3 extended variables including Utility, Anxiety, and Trust.

### UTAUT2

Venkatesh and Morris [32] proposed the Unified Theory of Acceptance and Use of Technology (UTAUT) to reveal the usage behavior of information technology systems. This theory is based on eight theories: rational behavior theory, technology acceptance model, innovation diffusion theory, planned behavior theory, TPB and TAM combination, personal computer usage model, motivation model, and social cognition theory. This theory believes that performance expectancy, effort expectancy, social Influence, facilitating conditions are all factors that directly affect user intention and are also influencing factors of usage behavior. Subsequently, Venkatesh and Thong [35] expanded UTAUT by incorporating three variables: hedonic motivation, price value, and habit. UTAUT has become one of the important theoretical models used to predict consumer technology use [35], and its effectiveness has been proven in different cultural backgrounds [36]. UTAUT2 extends the applicability of UTAUT from the overall organizational environment to the individual consumer environment [42]. We believe that UTAUT2 is more suitable for the context of this study as it was specifically developed based on the user’s perspective [35]. Therefore, this study is based on UTAUT2 theory.

#### Performance expectancy

Martins et al. [56] defined performance expectancy as the extent to which users perceive that adopting a particular technology will benefit their work, including advantages such as time savings and improved efficiency. When a technology system brings benefits to users, it encourages their adoption of the technology [57]. Lu and Su [51] indicated that performance expectancy has an impact on users’ adoption of mobile services. In previous research on user decision-making, it has been consistently confirmed that performance expectancy directly influences user behavioral intentions. For example, in online shopping [58], live shopping [59], and mobile shopping [60]. In addition, previous studies have confirmed the positive correlation between effort expectation and users’ Internet service intention [61] and mobile payment intention [62], and whether this relationship can be supported in the mobile shopping situation of the elderly needs further verification. We believe that mobile phones provide convenience for older adults in shopping, which will increase their willingness to use mobile shopping. Thus, the following hypothesis is proposed:

**H1**: Performance expectancy is positively correlated with the older adults’ intention to engage in mobile shopping.

#### Effort expectancy

Effort expectancy refers to the extent to which users perceive a technology as easy to use [35]. Hew et al. [63] confirmed a significant positive correlation between effort expectancy and users’ intention to use mobile applications. Morosan [13] identified effort expectancy as an important predictor of American users’ willingness to adopt mobile purchasing for airline travel auxiliary services, However, for Chinese users, the existence of this predictive effect remains to be verified. Hanif et al. [60] confirmed the positive impact of effort expectancy on users’ intention to make online purchases. Moreover, the positive influence of effort expectancy on user shopping behavior has been demonstrated in online mobile gaming [64] and e-commerce [59]. Hassan et al. [65] pointed out in his research on online shopping behavior of Malaysian students that trying to anticipate positively affects users’ behavioral intention. However, whether there are consistent results for Chinese users, especially the Chinese elderly population, remains to be verified. This study posits that the intelligent features of smartphones simplify user operations, requiring minimal effort from older adults, which may increase their willingness to use smartphones. Therefore, the following hypothesis is proposed:

**H2**: Effort expectancy is positively correlated with older adults’ intention to engage in mobile shopping.

#### Social influence

Social influence refers to the extent to which users perceive that important individuals in their lives believe they should adopt a particular technological product or service [35]. Social influence operates by altering users’ belief structures [66] and eliciting responses to social pressures [67], ultimately leading users to adjust their beliefs [68]. In the context of consumer online shopping behavior, users’ mobile shopping behavior may be influenced by individuals such as family members, friends, and colleagues [69], and users may seek their opinions before engaging in mobile shopping [70]. Yang et al. [71] found positive effects of social influence on users’ intention to adopt mobile payment services. Escobar-Rodríguez and Carvajal-Trujillo [14], in their study on online ticket purchases for low-cost airlines among Spaniards, demonstrated the significance of social influence as a factor. In the study of mobile shopping in Pakistan by Hanif et al. [60], it is confirmed that Social influence is the factor that affects the mobile shopping intention of Pakistani users. This study suggests that older adults’ intention to engage in mobile shopping may also be influenced by the opinions of family members, friends, and significant individuals in their lives. Hence, the following hypothesis is proposed:

**H3**: Social influence is positively correlated with older adults’ intention to engage in mobile shopping.

#### Facilitating conditions

Venkatesh et al. [32] defined facilitating conditions as the resources that users possess to support the use of technology. It represents users’ belief that relevant organizations and technological facilities can facilitate their use of the system. Venkatesh, Thong, and Xu [35] noted that facilitating conditions are associated with the support and resources available to users when performing a particular technological behavior. Therefore, it is often considered a driving factor for users’ intention to use technology and their actual usage behavior. For example, in Momani et al.’s [72] study on social commerce among Malaysian users, convenience conditions were found to have significant positive effects on both the intention and actual usage of social commerce behavior among Malaysian users. Hanif et al. [60] confirmed the positive impact of facilitating conditions on users’ behavior intention and actual usage behavior. Lallmahomed et al. [73] suggested that if support for a particular technology or system knowledge is available, users are more likely to have a higher intention to adopt the technology, which in turn influences users’ adoption of the technological system. This study believes that elderly users need to have relevant knowledge and resources when using smart phones to shop, such as mobile phone networking, shopping application installation knowledge and external assistance guidance. Therefore, when facilitating conditions are available, it may help to reduce the barriers for the elderly to adopt mobile shopping, thus leading to an increase in the intention and actual behavior of mobile shopping. Therefore, this study proposes the following hypothesis:

**H4**: Facilitating conditions is positively correlated with older adults’ intention to engage in mobile shopping.
**H5:** Facilitating conditions is positively correlated with older adults’ actual usage behavior of mobile shopping.

#### Hedonic motivation

Hedonic motivation is defined as the enjoyment and pleasure that users perceive during the process of using technology [35]. Bridges and Florsheim [74] suggest that hedonic experiences can be perceived enjoyment during the shopping experience. Mobile shopping platforms offer hedonic and non-shopping functional value to enhance users’ online shopping experience [70], and consumers are often influenced by hedonism in their decision-making [24]. Ener et al. [49] believed that consumers often shop for fun, not for the purpose of completing the shopping task itself. Kim et al. [75] pointed out that those users who experience pleasure from using mobile apps are more likely to adopt mobile apps. Hedonic factors have been considered as motivational drivers in studies on social networking sites [76], mobile payments [77], and other contexts. Falode et al. [78] argue that shopping is a delightful experience that elicits positive emotions and satisfaction from users. Previous research [79, 80] has demonstrated a positive relationship between hedonic motivation and users’ intention to engage in technological behaviors. In this study, hedonic value is defined as the enjoyable experience that older adults derive from mobile shopping. We hypothesize that the higher the perceived pleasure older adults derive from using mobile shopping, the higher their intention to engage in mobile shopping. Therefore, we propose:

**H6:** Hedonic motivation is positively correlated with older adults’ intention to engage in mobile shopping.

#### Price value

Al-Adwan et al. [28] pointed out that the value of m-commerce perceived by users is different. Price value is defined as the trade-off between the benefits users perceive from adopting a certain technology and its monetary costs [81]. Zhou et al. [59] point out that price and cost are important balancing concepts in users’ minds, as they influence users’ perception of product value. Some studies [14, 64] have identified price value as a motivational variable and confirmed its significant impact on users’ adoption of new technologies and applications. For example, in Liu et al.’s study [82] with Chinese respondents, it was found that perceived value has a positive influence on users’ intention to use mobile coupon applications. Turban and King [83] confirmed the influence of price on users’ online purchase intention. Chopdar and Sivakumar [84] point out that price value positively influences the persistent intention of users of mobile shopping applications. With the presence of discounts and coupons in online shopping, it has attracted user segments in emerging markets [85]. Al-Adwan et al. [28] believe that the difference between consumers’ value perception of e-commerce and mobile commerce is mainly reflected in risk, personalization and convenience, all of which have a significant impact on users’ business intention. However, Natarajan et al. [86] noted that price is given less attention in technology adoption. Additionally, some studies have failed to demonstrate the impact of price value on users’ behavioral intentions. For instance, Zhou et al. [59] on Chinese consumers’ adoption of e-commerce did not find a significant effect of price value on users’ behavioral intentions. Warganegara and Babolian Hendijani [87] examined the driving factors behind online food and grocery purchasing behavior among Indonesian users during the COVID-19 pandemic and found that price did not influence their intention to use. Therefore, investigating price value as a potential and underexplored variable is necessary and meaningful [87]. In summary, the effects of price value are not uniform, in the context of mobile shopping, it is important to explore the impact of price value on the older adult population. Thus, this study proposes the following hypothesis:

**H7**: Price value is positively correlated with older adults’ intention to engage in mobile shopping.

#### Habit

Habit is the feedback of past experiences and to some extent reflects the outcomes of those experiences [35]. It has an influence on users’ intention and actual usage behavior of new technologies [59, 88]. As Ajzen [89] pointed out, users’ past behavioral patterns are one of the important determinants of their current behavior. Kim [90] found that habit significantly affects the actual usage of mobile data services and applications. Hew et al. [63] conducted a study in Malaysia and identified habit as the strongest predictor of users’ intention to use mobile applications. Furthermore, previous research by Van Droogenbroeck and Van Hove [91] has demonstrated the significant impact of habit on users’ intention and actual usage of online grocery shopping. In the traditional shopping mode, consumers mostly shop in offline physical stores, which attract a large number of consumers, especially elderly users. However, compared with the traditional shopping mode, mobile shopping expands the time and scene of users’ shopping, which may change consumers’ shopping habits. Due to the strong concept and habit of the elderly, it may have an impact on the use of mobile shopping behavior. Therefore, based on previous studies, this study proposes::

**H8**: Habit is positively correlated with older adults’ intention to engage in mobile shopping.
**H9**: Habit is positively correlated with older adults’ actual usage behavior of mobile shopping.

#### Behavioral intention

Behavioral intention is defined as the strength of an individual’s intention to perform a specific behavior [92]. The actual behavioral performance of users depends on the intensity of their behavioral intention [93]. Therefore, behavioral intention can be used to predict users’ actual behavior [94]. In the UTAUT framework, behavioral intention is a precursor variable to actual usage behavior. Previous studies [59, 95] confirmed the strong positive effect of user behavioral intention on usage behavior. Gross [26] has pointed out that users’ mobile shopping behavior is influenced by their behavioral intention. Chopdar et al. [96] also confirmed the direct positive influence of consumer behavioral intention on actual usage behavior in their study on shopping mobile applications with respondents from the United States and India. Shaouf and Lu [97] pointed out that consumers’ purchase intention has a positive impact on their actual purchase behavior. Based on the above, this study also posits that the behavioral intention of older adults towards mobile shopping will positively influence their actual usage behavior. Therefore, the following hypothesis is proposed:

**H10**: Behavioral intention of older adults towards mobile shopping is positively correlated with actual usage behavior.

### Extended variables

#### Utilitarian

Maduku and Thusi [50] interpret the utilitarian value in the context of mobile shopping as the assessment of functional benefits that users perceive from mobile shopping. Utilitarian users focus on useful and behaviorally relevant aspects [98]. For instance, they consider the convenience of purchasing through mobile devices (such as freedom from time and location constraints) and the potential cost-saving benefits associated with shopping from online retailers using mobile devices. Therefore, utilitarian factors encompass multiple aspects, including economic costs [99] and convenience [50]. As early as 1972, Tauber [100] conducted research on user consumption motives and highlighted that users engage in the shopping process to attain intrinsic utilitarian value and gratification. Babin and Darden [101] pointed out that in addition to hedonism, utilitarianism also has an important impact on consumer behavior. However, the extent to which these consumer values explain users’ mobile shopping behavior has not received enough attention [50]. Utilitarianism plays a crucial role in distinguishing between online and offline consumption for users [102]. The utilitarian value is significant for mobile shopping [103]. As Kautish and Sharma [104] pointed out, the majority of users choose mobile shopping based on utilitarian reasons such as time savings and convenience of service. Thus, this study proposes:

**H11**: Utilitarianism is positively correlated with the elderly’s intention to shop using mobile phones.

#### Anxiety

Bandura [105] defined anxiety as the inclination of users to experience technological fear or concerns regarding a particular innovative technology or service [106]. Users should strive to avoid technology usage behaviors driven by anxiety [107], especially when facing mobile shopping technologies with potential issues [51].Bahli and Benslimane [108] mention that consumer anxiety about using data mobility services is higher in a mobile environment, as liability for transaction failure or loss may not be clear in such a technology-mediated environment. Users may worry about potential problems such as personal data leakage after engaging in mobile shopping [51]. They believe that mobile shopping could lead to monetary losses and information compromise, thus increasing their anxiety [109]. Previous studies [51, 110] have examined anxiety as a factor influencing user adoption of mobile shopping. Anxiety is an individual’s emotional barrier, negatively associated with their technology usage [51]. Lu and Su [51] believed that users’ anxiety would reduce their behavior of using mobile shopping websites. McFarland and Hamilton [111] point out that technological anxiety is a negative factor affecting user acceptance of information technology. According to the research of Yang and Forney [25], users’ anxiety about the adoption of mobile shopping is largely due to its novelty and uniqueness in shopping experience. Considering the highly personalized nature of mobile devices, concerns about privacy and security are evident. Therefore, investigating users’ negative reactions and their impact on technology adoption is an important issue [51]. In this study, anxiety refers to the negative emotions experienced by elderly individuals during the process of using mobile phones for mobile shopping [51]. The following hypothesis is proposed:

**H12**: Anxiety is positively correlated with the elderly’s intention to engage in mobile shopping using smartphones.

#### Trust

Alalwan, Dwivedi, and Rana [7] define trust as the subjective evaluation of users regarding the credibility of a particular technological product. It can be understood as a sense of security and represents the willingness of users to rely on someone or a particular technology [112]. Yang [113] states that trust is beneficial and important for reducing user uncertainty and increasing their sense of security. It significantly reduces external threats and influences consumers’ intention to engage in mobile purchases [114]. Hanafizadeh et al. [115], in their study on the adoption of mobile banking, found that trust is a preeminent motivating factor influencing user behavior. Wu and Chen [116] confirmed that users’ trust belief in technology is an important driving factor affecting users’ mobile shopping. Al-Adwan et al. [33] took the e-commerce customers in Jordan as the research object and examined the signals based on e-retailers. The results showed that customers’ trust in e-commerce would positively affect their online purchase intention. Furthermore, Nagy and Hajdú [117] investigated the acceptance of artificial intelligence in online shopping among Hungarian users, and the results of an online survey demonstrated that trust is a crucial factor influencing Hungarian users’ attitudes toward artificial intelligence. Trust impacts users’ purchase intentions and is an essential component for long-term brand loyalty in the context of technology adoption [70]. Additionally, user trust plays a vital role in improving acceptance levels of new technologies for online shopping and in the marketing promotion of merchants’ products [118]. The research of Al-Adwan and Al-Debei [29] reveals that users’ trust has a positive impact on customers’ re-purchase intention in e-commerce. Habib and Hamadneh [70] point out that because e-commerce is a virtual transaction, it needs users’ trust more than offline transactions. Despite previous research demonstrating the role of trust in user intentions, it remains of great importance, yet understudied, for the elderly user group in the context of mobile shopping. In this study, trust is defined as the perception of personal privacy security and the security of mobile shopping services among elderly individuals when using mobile phones for shopping. The following hypothesis is proposed:

**H13**: Trust is positively correlated with the elderly’s intention to shop using mobile phones.

In summary, this study provides a model for the acceptance of mobile shopping by the elderly, as shown in Fig 1.

## Methods

### Questionnaire design

This study employed a questionnaire to collect data. The researchers developed the questionnaire by referencing previous literature in line with the study’s topic. The questionnaire consisted of two parts: Part 1 included scales, and Part 2 included demographic questions. The scales comprised 12 constructs, totaling 37 measurement items. Among them, Performance Expectancy and Effort Expectancy were adapted from Venkatesh et al. [32], Social Influence was adapted from Venkatesh, Thong, and Xu [35] and Venkatesh et al. [32], the three measurement items for Facilitating Conditions were adapted from Venkatesh et al. [32] and Lian and Yen [21], the Hedonic Motivation construct drew upon Venkatesh, Thong, and Xu [35] and Habib and Hamadneh [70], the measurement indicators for Price Value, Habit, and Intention to shop using mobile phones were derived from Venkatesh, Thong, and Xu [35], the Utilitarian items were adapted from Lavuri et al. [119] and Lavuri, Kaur, and Thaichon [15], the Anxiety items were adapted from Saprikis et al. [8], the Trust items were adapted from Zhou et al. [59], and the Use Behavior items were adapted from Warganegara and Babolian Hendijani [87] and Zhou et al. [59]. Based on the previous literature mentioned above, the researchers designed a Chinese version of the questionnaire; Then translated into English by language experts proficient in both English and Chinese; Finally, the researchers and language experts checked whether the Chinese version of the questionnaire used in the survey was the same as the English version and whether it was suitable for the theme of this study, and the results were satisfactory. The questionnaire utilized a 7-point Likert scale ranging from 1 (strongly disagree) to 7 (strongly agree). Part 2 of the questionnaire included questions on gender, age, level of education, mobile phone usage experience, and mobile shopping experience in the past year, among others.

### Respondents and data collection

This study focused on Chinese elderly individuals who were aged 60 and above, capable of independent mobility, and had experience in mobile shopping. The researchers utilized purposive sampling to recruit participants from residential communities. These communities include old residential areas or new residential communities in urban areas. Firstly, researchers select respondents who may meet the age requirements based on their appearance characteristics, and consider them as potential respondents after receiving a clear response. Then, the elderly respondents were informed about the theme and objectives of the study. After obtaining their verbal consent and confirming their experience in mobile shopping, paper-based questionnaires were distributed to them. Some of the older people with poor vision filled out the questions with the help of a researcher reading the questions. Of course, this study attaches great importance to ethical issues, so the questionnaire was filled in anonymously, and no information concerning individuals and their privacy was collected during the research process. After knowing the purpose and content of the study, all participants orally agreed to participate in the study, and finally completed the questionnaire. In addition, the questionnaire research academic ethics application was approved by the academic committee of Guangdong Ocean University. A total of 411 questionnaires were collected for the study, and after eliminating invalid responses, 389 valid questionnaires remained. In this study, partial least squares structural equation modeling was used, and the sample size required 30–100 small samples [120], while Kline [121] believed that at least 200 samples were required. In addition, Kline [122] believes that the number of respondents is 5–10 times the number of questions measured in the questionnaire. To sum up, the 389 valid questionnaires in this study exceeded the requirement by more than ten times for the 37 measurement items, so the study sample size meets the requirements of the above scholars. The data was collected between April and May 2023.

### Statistical analysis

This study employed IBM SPSS Statistics 25 for descriptive statistical analysis, among other procedures. Simultaneously, considering the suitability of partial least squares structural equation modeling for prediction and theory development [123], as well as for complex models with multiple constructs [124]. Moreover, partial least squares structural equation modeling does not strictly require the assumption of normal distribution in the research data [125], that is, it does not require the sample data to meet the normal distribution hypothesis [126, 127]. Therefore, taking into account the number of constructs in the study model and other factors, this study utilized partial least squares structural equation modeling for data analysis. It mainly includes normality test, common method bias, reliability and validity, Goodness of Fit, standardized root mean square residual, and forecast correlation Q^2^ value.

## Results

### Demographic statistics

A total of 389 valid questionnaires were collected from the participants in this study, and their demographic characteristics are presented in Table 1. Among the 389 respondents, there were 218 male elderly individuals and 171 female elderly individuals. The age group of 60~64 years had the highest representation with 130 respondents, accounting for 33.4% of the total sample, followed by the age group of 65~69 years, which consisted of 115 individuals. Approximately 37.5% of the respondents reported having an educational level of elementary school or below, while there were 19 respondents with a university education or higher. Regarding mobile phone usage experience, 34.2% of the respondents had 1~3 years of experience, and the highest proportion belonged to the group with 4~6 years of experience, accounting for nearly half of the total sample. In terms of the frequency of mobile shopping in the past year, 31.1% of the respondents indicated a frequency of 6~9 times, and 41.4% of the respondents reported having more than 10 experiences of mobile shopping.

**Table 1:** Demographic characteristics.

Demographic	Characteristic	Counts	Percentage (%)
Gender	Male	218	56.0
	Female	171	44.0
Age	60~64	130	33.4
	65~69	115	29.6
	70~74	72	18.5
	75~79	55	14.1
	≥80	17	4.4
Education	Elementary school or lower	146	37.5
	Junior high school	136	35.0
	High school	88	22.6
	University education or higher	19	4.9
Mobile phone usage experience	1~3 years	133	34.2
	4~6 years	183	47.0
	7~9 years	52	13.4
	≥10 years	21	5.4
Mobile shopping experience in the past year	1~5 times	107	27.5
	6~9 times	121	31.1
	10~14 times	77	19.8
	15~19 times	53	13.6
	≥20 times	31	8.0

### Normality test

The structural equation model requires the data to be normally distributed, so the normal distribution test is first carried out in this study. Bollen and Long [128] believe that whether the observed variables conform to the normal distribution can be assessed by measuring the skewness coefficient and kurtosis coefficient of the distribution of variables. Moreover, when the absolute values of skewness and kurtosis of the observed variables are both less than 2, the observed variables can be identified as normal. The sample data in this study conform to normal distribution, and the results are shown in Table 2.

**Table 2:** Normal distribution test results.

Items	Indicator	Mean	Skewness	kurtosis
PE	PE1	4.51	0.165	-0.052
	PE2	4.49	0.033	-0.392
	PE3	4.75	-0.366	-0.190
EE	EE1	4.57	-0.462	0.240
	EE2	4.70	-0.510	0.309
	EE3	5.06	-0.918	0.915
	EE4	4.70	-0.151	0.117
SI	SI1	5.21	-0.302	-0.315
	SI2	5.37	-0.492	-0.175
	SI3	5.22	-0.331	-0.402
FC	FC1	4.22	-0.159	0.291
	FC2	4.24	0.093	0.104
	FC3	4.28	-0.162	0.281
HM	HM1	4.52	0.039	-0.121
	HM2	4.56	0.124	-0.343
	HM3	4.54	-0.233	-0.044
PV	PV1	4.81	-0.569	0.460
	PV2	4.82	-0.549	0.436
	PV3	4.75	-0.587	0.624
HA	HA1	4.90	-0.236	0.083
	HA2	4.98	0.079	-0.482
	HA3	4.91	0.240	-0.666
BI	BI1	4.87	-0.330	-0.610
	BI2	4.80	-0.353	-0.271
	BI3	4.64	-0.115	-0.592
UB	UB1	4.89	-0.255	-0.106
	UB2	4.86	-0.260	0.323
	UB3	4.71	-0.129	0.038
UT	UT1	4.81	-0.210	0.281
	UT2	4.91	0.094	-0.369
	UT3	4.85	0.426	-0.628
AN	AN1	5.14	-0.267	-0.339
	AN2	5.34	-0.523	-0.141
	AN3	5.17	-0.390	-0.192
TR	TR1	4.55	0.076	-0.163
	TR2	4.61	0.034	-0.393
	TR3	4.74	-0.293	-0.274

Note: AN: Anxiety; EE: Effort expectancy; FC: Facilitating conditions; HA: Habit; HM: Hedonic Motivation; PE: Performance expectancy; PV: Price Value; IN: Mobile shopping behavioral intention; SI: Social influence; TR: Trust; UB: Use behavior; UT: Utilitarian.

### Common method bias

The research questionnaire utilized simplified language for item descriptions [5] and was administered anonymously to mitigate common method bias. Given that the research data were collected using a questionnaire, the potential issue of common method bias needed to be addressed. Therefore, after the completion of the questionnaire, a Harman’s single-factor test was conducted following the recommendations of Podsakoff et al. [129]. The results revealed that the largest single factor accounted for only 23.089% of the total variance, which was below the threshold of 50%, indicating the absence of common method bias [2, 130]. In addition, we are not limited to using only one method to assess common method bias. Kock [131] believes that common method bias does not exist when the variance inflation factor is less than or equal to 3.3. In this study, all VIF values (as shown in Table 3) range from 1.012 to 1.598, indicating that there is no common method bias in this model.

**Table 3:** Variance inflation factor.

	Smartphone shopping intentions	Use behavior
Anxiety	1.029	
Effort expectancy	1.568	
Facilitating conditions	1.550	1.476
Habit	1.559	1.441
Hedonic motivation	1.016	
Performance expectancy	1.598	
Price value	1.012	
Smartphone shopping intentions		1.569
Social influence	1.178	
Trust	1.016	
Use behavior		
Utilitarian	1.038	

### Measurement model

The measurement model was evaluated in this study through reliability, discriminant validity, and convergent validity assessment [132]. The results of the reliability and validity evaluation are presented in Tables 4 to 6. For reliability assessment, the criteria of Cronbach’s alpha greater than 0.7, composite reliability (CR) greater than 0.7, and average variance extracted (AVE) greater than 0.5 can be used [133]. As shown in Table 2, all reliability indicators meet the threshold, indicating good reliability. The evaluation of validity can be conducted through convergent validity and discriminant validity. When the AVE value exceeds 0.50, it indicates the model has convergent validity [134]. As shown in Table 4, the AVE values for all constructs exceed 0.5, indicating good convergent validity of the model. Fornell and Larcker [135] suggested that when the standardized factor loadings of the measurement items are greater than 0.5, it indicates that the measurement items adequately explain the latent variables, demonstrating satisfactory indicator reliability [136]. Table 4 displays that all items have loadings that meet the criterion. Additionally, the Fornell-Larcker criterion [135] and Heterotrait-Monotrait Ratio (HTMT) values [137] are indicators of discriminant validity. According to the Fornell-Larcker criterion, if the square root of the AVE value for each construct is greater than the correlation coefficient between constructs, it indicates acceptable discriminant validity of the questionnaire [135]. Table 5 shows that the square root of AVE values along the diagonal meet the criterion, indicating good discriminant validity of the model. Furthermore, Henseler, Ringle, and Sarstedt [137] suggest that the Heterotrait-Monotrait Ratio (HTMT) can be used to assess discriminant validity between constructs. When the HTMT value is less than 0.9, it signifies discriminant validity among constructs [138]. Table 6 displays that all HTMT values are less than 0.9, indicating the achievement of the discriminant validity criterion. In summary, the model exhibits satisfactory reliability and validity.

**Table 4:** Construct reliability and validity.

Items	Indicator	Loadings	Mean	Cronbach’s Alpha	Composite Reliability	Average Variance Extracted (AVE)
AN	AN1	0.830	5.2151	0.824	0.892	0.733
	AN2	0.830				
	AN3	0.907				
EE	EE1	0.811	4.7577	0.846	0.896	0.683
	EE2	0.848				
	EE3	0.816				
	EE4	0.831				
FC	FC1	0.925	4.2442	0.878	0.925	0.804
	FC2	0.926				
	FC3	0.836				
HA	HA1	0.909	4.9314	0.903	0.939	0.837
	HA2	0.922				
	HA3	0.914				
HM	HM1	0.912	4.5416	0.903	0.938	0.835
	HM2	0.942				
	HM3	0.885				
PE	PE1	0.792	4.5827	0.816	0.889	0.728
	PE2	0.871				
	PE3	0.892				
PV	PV1	0.933	4.7952	0.873	0.919	0.790
	PV2	0.859				
	PV3	0.872				
IN	IN1	0.897	4.7729	0.866	0.918	0.788
	IN2	0.885				
	IN3	0.881				
SI	SI1	0.814	5.2648	0.804	0.883	0.716
	SI2	0.865				
	SI3	0.859				
TR	TR1	0.916	4.6341	0.893	0.933	0.823
	TR2	0.899				
	TR3	0.908				
UB	UB1	0.839	4.8201	0.813	0.889	0.728
	UB2	0.859				
	UB3	0.861				
UT	UT1	0.902	4.8595	0.890	0.931	0.818
	UT2	0.921				
	UT3	0.890				

**Table 5:** Fornell-Larcker criterion.

Construct	AN	EE	FC	HA	HM	PE	PV	IN	SI	TR	UB	UT
AN	**0.856**											
EE	-0.044	**0.827**										
FC	0.019	0.469	**0.897**									
HA	0.035	0.412	0.456	**0.915**								
HM	-0.006	0.026	-0.034	0.009	**0.914**							
PE	-0.096	0.506	0.460	0.442	-0.071	**0.853**						
PV	0.012	0.022	0.068	0.035	0.003	0.059	**0.889**					
IN	-0.109	0.505	0.522	0.505	-0.069	0.473	0.047	**0.888**				
SI	-0.057	0.146	0.257	0.359	-0.048	0.163	0.033	0.330	**0.846**			
TR	-0.049	0.083	0.046	0.054	0.026	0.087	-0.046	0.130	0.042	**0.907**		
UB	-0.024	0.525	0.445	0.492	-0.022	0.423	-0.016	0.573	0.227	0.178	**0.853**	
UT	0.002	0.149	0.114	0.031	0.004	0.015	-0.044	0.151	0.003	-0.001	0.028	**0.905**

**Table 6:** Heterotrait-Monotrait ratio.

Construct	AN	EE	FC	HA	HM	PE	PV	IN	SI	TR	UB	UT
AN												
EE	0.052											
FC	0.090	0.532										
HA	0.062	0.465	0.509									
HM	0.050	0.048	0.044	0.030								
PE	0.112	0.587	0.527	0.507	0.080							
PV	0.041	0.056	0.074	0.046	0.008	0.057						
IN	0.123	0.580	0.593	0.571	0.076	0.548	0.050					
SI	0.073	0.167	0.297	0.416	0.060	0.183	0.064	0.389				
TR	0.061	0.096	0.052	0.059	0.044	0.107	0.067	0.147	0.052			
UB	0.064	0.628	0.519	0.572	0.029	0.505	0.061	0.683	0.269	0.207		
UT	0.045	0.170	0.129	0.043	0.015	0.056	0.063	0.168	0.055	0.035	0.038	

In addition, before structural model analysis, multi-collinearity tests should be carried out to prevent collinearity problems from affecting the evaluation of model path coefficients. According to Kock and Lynn [139], variance inflation factor (VIF) is used as an index to evaluate the collinearity problem. When VIF is less than 3.3, the collinearity problem is not serious. Table 3 shows that the VIF values in this study range from 1.012 to 1.598, both of which are less than 3.3. This shows that the collinearity problem does not have a negative impact on the evaluation of structural models.

### Structural model

In terms of model fit, this study assessed the Goodness of Fit (GOF) and Standardized Root Mean Square Residual (SRMR). GOF is an effective measure to evaluate the adequacy of the model, with a GOF value exceeding 0.36 indicating good model fit [140]. The calculated Goodness of Fit in this study is 0.578, surpassing the threshold of 0.36 for high model fit, indicating a high level of fit. Additionally, Henseler et al. [141] suggested using standardized root mean square residual (SRMR) to assess the overall model fit. When the SRMR value is less than 0.08, it indicates good model fit [142]. The result of this study shows an SRMR value of 0.046, meeting the criteria for good model fit. In summary, the research model demonstrates good fit. In addition, Hair and Risher [140] pointed out that in the structural model, the predictive correlation Q^2^ value of the intrinsic dimension plane is greater than 0, indicating that the structural model has predictive correlation for the reflective intrinsic construct; and when Q^2^ is higher than 0, 0.25 and 0.50, it indicates that the partial least squares path model has small and large predictive correlation, respectively. The Q^2^ values of Smartphone shopping intentions and Use behavior constructs in this study were 0.358 and 0.283, respectively, indicating that the model has a moderate predictive correlation.

**Fig 2:**
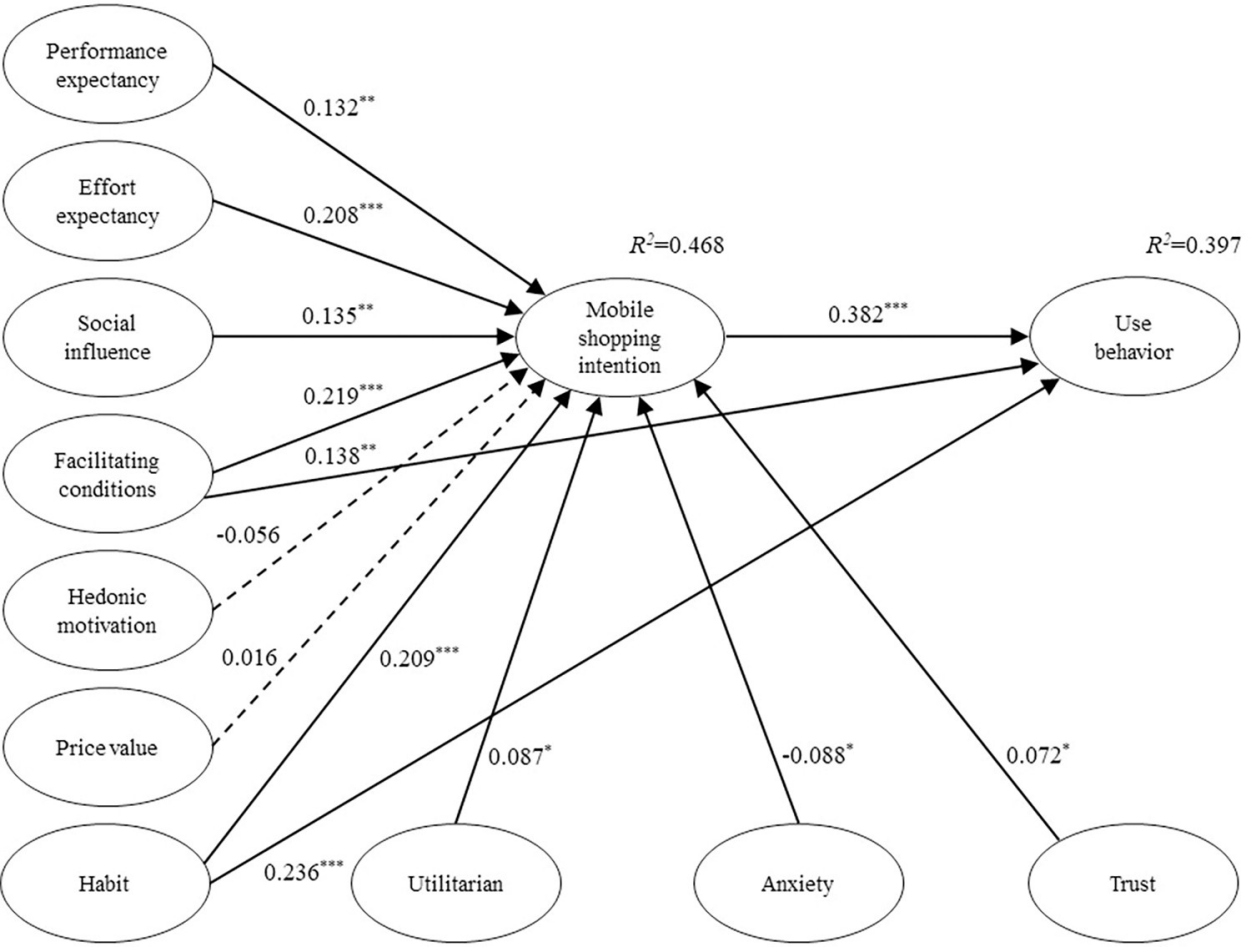
Model evaluation result.

According to the recommendation by Hair et al. [143] regarding 5000 bootstrap resampling, the significance of path coefficients and hypotheses were evaluated, as shown in Fig 2 and Table 7. Among the 13 proposed research hypotheses, the majority were supported, with only two hypotheses not being confirmed. As shown in Table 7, performance expectancy (β = 0.132, t = 2.584, p<0.01), effort expectancy (β = 0.208, t = 4.769, p<0.001), social influence (β = 0.135, t = 3.152, p<0.01), facilitating conditions (β = 0.219, t = 4.329, p<0.001), and habit (β = 0.209, t = 4.464, p<0.001) directly influence the smartphone shopping intentions of older adults. Therefore, H1, H2, H3, H4, and H8 are supported. Moreover, facilitating conditions (β = 0.138, t = 2.756, p<0.01), habit (β = 0.236, t = 4.311, p<0.001), and smartphone shopping intentions (β = 0.382, t = 6.836, p<0.001) also have a direct significant positive impact on use behavior. Hence, H5, H9, and H10 are confirmed. However, in our study, hedonic motivation (β = -0.056, t = 1.419, p = 0.156) and price value (β = 0.016, t = 0.361, p = 0.718) do not have a significant direct influence on smartphone shopping intentions of older adults, thus H6 and H7 are not supported. Additionally, utilitarian (β = 0.087, t = 2.242, p<0.05), anxiety (β = -0.088, t = 2.225, p<0.05), and trust (β = 0.072, t = 2.031, p<0.05) also directly impact the smartphone shopping intentions of older adults, supporting H11, H12, and H13.

**Table 7:** Hypotheses results.

Hypotheses	Path	β	T	P Values	Supported
H1	PE → IN	0.132	2.584	0.010**	Yes
H2	EE → IN	0.208	4.769	0.000***	Yes
H3	SI → IN	0.135	3.152	0.002**	Yes
H4	FC → IN	0.219	4.329	0.000***	Yes
H5	FC → UB	0.138	2.756	0.006**	Yes
H6	HM → IN	-0.056	1.419	0.156	No
H7	PV → IN	0.016	0.361	0.718	No
H8	HA →IN	0.209	4.464	0.000***	Yes
H9	HA→ UB	0.236	4.311	0.000***	Yes
H10	IN → UB	0.382	6.836	0.000***	Yes
H11	UT→ IN	0.087	2.242	0.025*	Yes
H12	AN →IN	-0.088	2.225	0.026*	Yes
H13	TR→ IN	0.072	2.031	0.042*	Yes

Note

*, p < 0.05

**, p < 0.01

***, p < 0.001.

## Discussion and contribution

### Discussion

The purpose of this study is to propose a comprehensive model to understand the intention and usage behavior of older adults in smartphone shopping. Structural equation modeling was employed for analysis, revealing the influences of utilitarian, anxiety, trust, performance expectancy, effort expectancy, social influence, facilitating conditions, and habit on the smartphone shopping intentions and use behavior of older adults. This study represents an extension of the UTAUT2 theory and its application in the context of older users. It provides a theoretical basis for investigating the smartphone shopping behavior of older users and offers insights for the age-friendly design and further adoption of mobile shopping.

In the study of Keszey [144], utilitarianism has been regarded as an important variable in determining users’ purchase intention. Similarly, our research results validate the positive effect of utilitarian on user intentions. In other words, when users perceive more benefits and advantages from smartphone shopping, their intentions to engage in such behavior become stronger. This finding is consistent with the research conducted by Tam et al. [145]. The older respondents in this study may choose to use smartphone shopping for the sake of convenience and cost-saving considerations, as mobile devices offer flexibility, mobility, personalization, and other advantages [4]. Many businesses provide discounts or coupons to consumers who make purchases through mobile applications. After all, as highlighted by Kautish and Sharma [104], most users opt for mobile shopping based on utilitarian reasons such as saving time and convenience. Therefore, providers of smartphone shopping services should carefully consider utilitarian factors, gain insight into users’ utilitarian motivations, leverage the advantages of mobile devices, strengthen and improve user benefit mechanisms, and meet a range of utilitarian needs such as cost-saving, convenience, comfort, and product variety, in order to enhance users’ intention to engage in smartphone shopping. At the same time, online consumers usually pay attention to their utilitarian motives, and they prefer to grasp the quality of products and related after-sales service [146]. To this end, for the elderly, online mobile shopping platforms and product sellers should also strive to improve their products and services when providing customers with benefits such as coupons and discounts.

Additionally, Our research shows that Anxiety has a significant negative impact on the smartphone shopping intentions of older users. This finding is consistent with the results of McFarland and Hamilton [111] and Lu and Su [51]. Among them, Lu et al. [51] pointed out that users’ anxiety is an emotional barrier to the use of innovative systems, and also a key negative factor affecting users’ willingness to use mobile shopping websites. Our findings suggest that older consumer respondents who experience lower levels of anxiety towards technological systems do not perceive difficulties in using such systems and are more willing to adopt them. McFarland and Hamilton [111] pointed out that user anxiety towards technology use primarily centers around their ability and willingness to use the technology. For example, in the context of mobile payments, users may worry about pressing the wrong button on their mobile device during the payment process, resulting in financial loss [51]. Therefore, efforts should be made to reduce the complexity of smartphone shopping services to alleviate the concerns and anxieties of older users regarding operational errors, thereby increasing their perceived self-confidence in their own operational abilities. At the same time, developers of smartphone shopping applications should pay attention to product features that may trigger anxiety in older users and strive to optimize them to reduce user anxiety and enhance the shopping experience of older users. Of course, designers should reduce the features that may cause anxiety among older users, and technology platform providers also need to find ways to reduce the complexity of system services to reduce anxiety among older users.

Consistent with previous research, our study found that Trust has a positive impact on user intentions. Previous research has confirmed the important role of trust in users’ purchasing decisions. Wu et al. [116] confirmed that users’ trust beliefs in technology are important driving factors influencing their mobile shopping behavior. Hanafizadeh et al. [115], in their study on the adoption of mobile banking, found that trust is a preeminent motivating factor for user behavioral intentions. Trust, as an important concept, is often regarded as a key factor in user behavior in e-commerce environments [147] and requires sufficient attention. With the development of 4G network technology and the increase of smartphones, the speed and convenience of mobile shopping have increased, and users are enthusiastic about mobile shopping [15]. But mobile devices typically contain user personal data information [13]. In addition, many current mobile trading activities require a certain degree of mandatory licensing before proceeding, which increases users’ doubts about using mobile devices for transactions and their results [148]. So, considering that users may face some uncertainty in the process of mobile shopping [149], trust is beneficial and crucial in reducing user uncertainty and increasing their sense of security [113]. Therefore, enhancing users’ trust in smartphone shopping needs to be considered. As the hardware medium for smartphone shopping, mobile devices largely bear the trust of older users. Therefore, efforts should be made to improve information security, operational security, and brand quality of smartphones to increase the sense of security and trustworthiness of older users towards smartphones. In addition, for mobile shopping applications, businesses should provide older users with sufficient security measures, reduce unnecessary personal information permissions, mitigate potential uncertainty, and enhance older users’ sense of security and trust in the applications.

Moreover, our research results demonstrate that performance expectancy and effort expectancy have a positive impact on the smartphone shopping intentions of older adults. This finding has been validated in previous studies, including e-commerce shopping [59] and online grocery retailing [70]. Smartphone shopping is distinct from traditional brick-and-mortar shopping and traditional e-commerce as it expands the shopping scenarios and allows users to shop without limitations of time and location, saving time, and enhancing the quality of consumer experience [96]. Additionally, Yang [150] pointed out that users pay attention to the convenience of accessing and navigating mobile websites when engaging in mobile shopping. Therefore, as businesses such as smartphone brands or mobile shopping applications, it is important to leverage the convenience of smartphone mobility and enhance users’ perception of performance expectancy and effort expectancy. For example, video tutorials tailored to older adults can be provided through mobile shopping applications to assist users in their smartphone shopping experience. Alternatively, simplifying the steps involved in using the phone and making purchases, designing user-friendly interfaces, and providing guidance can enhance the usability and utility of smartphone shopping, enriching the shopping experience for older users. Furthermore, this study found that price value has no impact on users’ smartphone shopping intentions, which aligns with the findings of Zhou et al. [59]. At the same time, Hedonic motivation does not influence the smartphone shopping intentions of older adults. This finding is contrary to the findings of Akram and Junaid [55], which examined the relationship between hedonic and utilitarian motivations and online purchase intention in the Chinese social business environment. This difference may occur because these older individuals are more rational and prioritize the task of shopping itself, rather than seeking pleasure during the smartphone shopping process. Considering that pleasure comes from consumers’ high investment and experiential enjoyment during the shopping process [119], as well as the potential demand for psychological satisfaction among current users. Therefore, mobile shopping related platforms or developers can also try to enrich the emotional and enjoyable experience that mobile shopping brings to the elderly by designing services that meet their needs.

Meanwhile, this study confirms the positive influence of social influence on smartphone shopping intentions. This indicates that the opinions of important individuals in older adults’ lives, such as family members and friends, significantly affect their adoption of smartphone shopping. This finding aligns with previous research [60]. In addition, social influence was also identified as a key factor in the study of mobile shopping usage in India [151] and Pakistan [60]. Therefore, to enhance the adoption of smartphone shopping among older adults, in addition to focusing on the older adults themselves, we can also influence them by promoting positive evaluations from individuals surrounding them, thus influencing their attitudes. Additionally, our study demonstrates a positive impact of older adults’ smartphone shopping intentions on their use behavior, which has also been confirmed in previous research on mobile banking [152], e-commerce [59], and online shopping [153]. It can be said that previous studies [152] reinforce the finding in this study that behavioral intention has a positive impact on usage behavior.

Furthermore, our research findings reveal that users’ habits directly and positively influence their smartphone shopping intentions and behaviors. This finding is consistent with the results of Chopdar and Sivakumar [84]. It suggests that platforms related to smartphone shopping should fully consider the utility of habit factors and provide services that align with current technological developments and users’ consumption habits. Simultaneously, leveraging the convenience and other features inherent in smartphone shopping is crucial for shaping users’ shopping habits. That is to say, smartphones, shopping platforms, and merchants should collaborate in multiple ways to implement multi-level marketing communication strategies and create user habits of using mobile phones for online shopping. As mentioned by Habib et al. [70], consumers have already shifted their purchasing habits from traditional stores and shopping centers to online platforms. Therefore, platforms and businesses related to smartphone shopping should implement strategies to maintain and enhance user stickiness, comprehensively shaping users’ habits of smartphone shopping. For example, attracting consumers through reward mechanisms such as purchasing points redemption, discounts, and regular promotions can gradually foster users’ purchasing habits, thereby promoting widespread use of smartphone shopping. That is to say, mobile and online suppliers regularly engage in incentive models to attract users [59], and through incentive measures, encourage users to develop the habit of using mobile phones for shopping.

## Theoretical and practical implications

This study has some theoretical significance. First, this study extends the UTAUT theoretical model by incorporating Utilitarian, Anxiety, and Trust factors in the context of mobile shopping, confirming the applicability of the extended UTAUT model in researching mobile shopping among older adults. Second, this study proposes a model for the acceptance of mobile shopping by older adults and utilizes rich data and structural equation modeling to validate the model’s goodness of fit and explanatory power. This provides a theoretical foundation for researching older adults’ mobile shopping behavior and serves as a reference for studying user behavior among other user groups and technologies. Lastly, this research was conducted in China, the world’s largest consumer market, which differentiates it from previous studies that primarily focused on samples from developed countries [13, 14]. Simultaneously, this study focuses on the elderly population, expanding the UTAUT theoretical model’s applicability to a different user group from the previous focus on younger generations [15]. This enriches the understanding of older adult users and expands the scope of the UTAUT theoretical model.

In addition, this study also has some practical significance. First, this study provides insights for stakeholders in the field of mobile shopping. The results demonstrate that performance expectancy, effort expectancy, utilitarian, anxiety, and trust have a direct impact on the intention of older adults to engage in mobile shopping. Therefore, professionals in the mobile shopping industry, including companies and designers involved in the development of software and hardware, should prioritize older adult users and consider human-computer interaction and user needs throughout the mobile shopping process. This approach will enhance the usability and availability of mobile shopping software and hardware, increase older adults’ trust in mobile shopping, reduce their anxiety, and fulfill their utilitarian needs in mobile shopping.Second, the study validates the conceptual model proposed based on the UTAUT2 framework. The development and validation of this conceptual model can serve as a reference for practitioners and policy-making institutions in the mobile shopping industry. It can assist in formulating relevant marketing and management strategies to ultimately improve user adoption of mobile shopping. Thirdly, this study contributes to a better understanding of older adult users for mobile shopping retailers. It provides inspiration for the development, design, and marketing of age-friendly mobile shopping products, thereby promoting the use of mobile shopping and enhancing positive aging among older adults.

## Limitations and future work

In this study, there are still some limitations that should be addressed in future research. Firstly, the participants in this study were older adults from China, a developing country, which limits the generalizability of the research findings. Subsequent studies should expand the sample to include groups from different cultural backgrounds, which may yield different findings, at the same time, cross-regional comparative research is also worth exploring. Secondly, although this study identified the factors influencing older adults’ mobile shopping based on relevant theories, human behavior is complex, and motivations behind behavior may involve multiple factors. Therefore, future research should incorporate other potential factors to comprehensively understand the mechanism of older adults’ mobile shopping behavior. Third, despite the participants in this study having experience in using mobile phones and engaging in mobile shopping, over time and with increased user experience, individuals’ technological attitudes and behaviors may change. Therefore, the cross-sectional design of this study is also a limitation, and future research should incorporate longitudinal studies to account for these changes. Fourth, although UTAUT2 provides theoretical guidance for the exploration of user behavior, other applications in the field of mobile shopping for the elderly can still be explored and expanded in the future to enrich the theoretical system of mobile shopping behavior for the elderly. Finally, the use of mixed methods may enrich the research results, and qualitative research can also explore deeper psychological feelings of users. Therefore, the subsequent research can try to carry out research by a mixed method combining qualitative and quantitative methods, in order to show more comprehensive scientific findings.

## Conclusion

With the intensification of population aging, there is an urgent need to understand the factors influencing older adults’ use of mobile shopping. This study proposes an extended model that integrates Utilitarian, Anxiety, and Trust, based on the UTAUT2 framework, to investigate the mobile shopping behavior of older users. The research findings indicate that older adults’ intention to engage in mobile shopping is positively influenced by utilitarian, anxiety, trust, performance expectancy, effort expectancy, social influence, facilitating conditions, and habit. Furthermore, facilitating conditions, habit, and older adults’ intention to engage in mobile shopping significantly and positively impact use behavior. Therefore, this study expands the UTAUT2 theory and its application in the field of user behavior. The research provides important theoretical foundations and references for understanding the intentions and usage behavior of older adults in mobile shopping, contributing to the realization and widespread adoption of age-friendly mobile shopping and promoting positive aging among older adults.

## Supporting information

S1 File Research questionnaire data. (CSV)
